# Exploration of soil microbial community: a comparative study between organic and conventional systems in perennial and annual crops

**DOI:** 10.1038/s41598-026-49298-1

**Published:** 2026-05-14

**Authors:** Jackson Kawakami, Mateus Oliveira Gomes, Paulo Roberto Da-Silva, André Freire Cruz

**Affiliations:** 1 Midwestern State University of Paraná, UNICENTRO, Paraná, Brazil; 2 Kyoto Prefectural University, Kyoto, Japan

**Keywords:** Biomarker taxa, Conventional agriculture, Functional prediction, Microbial diversity, Organic farming, Soil microbiota, Ecology, Ecology, Environmental sciences, Microbiology

## Abstract

​Soil microbiota is central to agroecosystem sustainability, influencing fertility, plant health, and resilience. Agricultural practices shape these communities in distinct ways: conventional systems, with intensive chemical inputs, often promote microbial homogeneity, whereas organic systems, with higher organic matter inputs and reduced disturbance, tend to sustain functionally diverse microbiomes. Yet, the extent of these effects remains poorly understood, particularly when taxonomic, functional, and machine-learning–based indicators are evaluated jointly across different cropping systems. To address this gap, we compared the taxonomic and functional diversity of soil microbiota in organic and conventional fields cultivated with common bean (*Phaseolus vulgaris*) and grapevine (*Vitis vinifera*) in Brazil. Microbial communities were profiled using 16 S rRNA and ITS sequencing, with analyses spanning diversity metrics, taxonomic composition, functional inference, and machine-learning-based biomarker identification. Bacterial diversity was greater under conventional management, while fungal diversity did not differ between systems. Machine-learning approaches identified *Bradyrhizobium* and *Roseococcus* (bacteria) and *Lecythophora* (fungus) as consistent biomarkers of organic soils. Functional predictions pointed to enhanced nitrogen fixation and carbon cycling in organic fields, whereas sulfur cycling was more prominent in conventional ones. Microbial community structure clearly separated by management and correlated strongly with soil chemistry, and Venn analysis revealed more unique bacterial and fungal operational taxonomic units (OTUs) in organic soils. Together, these results highlight how agricultural management acts as a strong ecological filter of soil microbiota. Conventional farming favored broader bacterial diversity. In contrast, organic systems fostered distinct microbial biomarkers and enhanced functional potential, particularly for nitrogen and carbon pathways.

**Supplementary Information:**

The online version contains supplementary material available at 10.1038/s41598-026-49298-1.

## Introduction

Organic agriculture has gained global relevance as an agricultural management system that influences soil processes and ecosystem functioning, driven by mounting concerns over environmental sustainability, human health, and food security. Among its numerous benefits, organic management is particularly lauded for restricting or eliminating synthetic pesticides and fertilizers, which can consequently favor the environment. This reduces contamination and harmful residue accumulation in natural areas, contributing to greater ecosystem balance and biodiversity preservation compared to conventional farming systems^1,2^. Enhanced ecosystem services, from nutrient cycling to soil and water quality, are well-documented findings from organic systems^3–5^.

Brazil has solidified its position as a key player in organic agriculture, with Paraná state being a great leader in this system. The number of certified organic farmers in Paraná has grown steadily, with government and private programs driving expansion, and certification programs improving market access, sustainability, and food quality^6,7^. A central pillar of organic agriculture is the stewardship of soil health, which is deeply connected to the composition and diversity of the soil microbial community. Research shows organic management not only increases soil organic matter, carbon, and nutrient reserves, but also boosts the abundance and diversity of key microbial taxa^8,9^. For instance, soils under organic management typically exhibit richer communities of beneficial fungi and bacteria, such as mycorrhizal fungi and plant growth-promoting rhizobacteria, compared to conventional practices^10,11^. These microorganisms promote soil structuring, nutrient cycling, and pathogen suppression—processes that create positive feedback loops and contribute to ongoing ecosystem resilience^3,12^. Furthermore, studies demonstrated that the application of organic fertilizers can substantially increase microbial abundance, diversity, and the complexity of microbial networks and stability compared to conventional chemical fertilizers^12^.

Common bean (*Phaseolus vulgaris*, bean) is a staple food in Brazil, a country that ranks as one of the world’s leaders in both production and consumption^13^. Paraná is the top-producing state, with more than 2.7 million tons harvested in 2024, and family farmers make up the bulk of bean producers^6^. In this context, organic management is not only ecologically appropriate but also economically viable for small-scale farmers, given the crop’s importance to regional food security and rural livelihoods.

Grapevine (*Vitis vinifera*) cultivation, likewise, represents an important economic asset for Brazil’s agricultural sector, and particularly in Paraná. Grape is mostly grown in permanent systems with minimal tillage—this contrasts with bean production, which relies on regular tillage—creating differing impacts on soil microbial communities^11,14^. The impact of agricultural practices—particularly these types of tillage—on soil microbial communities is profound. Fungal populations, in particular, are known to suffer from tillage due to the mechanical disruption of hyphal networks, resulting in community and ecosystem function changes^12^. Tillage can break up fungal hyphae, reduce fungal abundance, and alter the structure of both bacterial and fungal populations, while no-tillage or reduced tillage (prevalent in perennial fruit growing systems such as grape orchards) supports more stable, fungi-rich microbial networks^11^.

Advancements in high-throughput metagenomic sequencing have revolutionized our ability to characterize soil microbial communities in detail, especially those microorganisms that cannot be cultivated in culture media. Metagenomic approaches yield broad, culture-independent insights into microbial diversity, taxonomic structure, and functional potential, linking management practices to shifts in soil biology in previously inaccessible ways^15,16^. This molecular understanding is crucial for not only scientific progress but also for educating farmers and stakeholders on the tangible long-term benefits of organic agriculture^12,16^.

Given the economic, social, and ecological importance of bean and grape cultivation in southern Brazil, gaining a clearer understanding of how organic and conventional management practices influence soil microbial communities is crucial. Accordingly, this study aimed to investigate how contrasting agriculture management systems selectively shape soil microbial communities, encompassing both bacteria and fungi, in soils cultivated with bean and grape under organic and conventional systems at several sites in the Center-South of Paraná, Brazil. Specifically, we sought to (i) compare microbial diversity and community composition between management systems, (ii) identify bacterial and fungal taxa associated with organic and conventional practices, and (iii) relate microbial patterns to soil chemical properties and management characteristics. By employing metagenomic methodologies, this research provides an integrated assessment of management-driven microbial selection rather than uniform diversity enhancement, supporting the advancement of sustainable and resilient agricultural practices for the region^9,11,15^.

## Methods

Detailed information regarding the study sites, cropping systems, management practices, and other relevant characteristics are presented in Table 1. Soil samples were collected from agricultural fields managed under different farming systems. Sampling was conducted on March 18 and 19, 2025. In each sampling unit, four soil samples were collected from 0 to 10 cm depth, directly within the sowing or planting row, at a midpoint equidistant from two neighboring plants.

**Table 1:** Field survey of bean and grape farmers under two cultivation systems in 6 areas of Paraná state, Brazil.

Management	Organic	Conventional	Organic	Conventional	Organic	Conventional	Organic	Conventional
Crop	Bean	Bean	Bean	Bean	Grape	Grape	Grape	Grape
Municipality	Pinhão	Candói	Reserva do Iguaçu	Candói	Guarapuava	Guarapuava	Bituruna	Bituruna
Cultivar	unknown (black bean)	IPR Urutau	IPR Tuiuiú	IPR Esteio	Sangiovese	Sangiovese	Terci	Terci
Area (ha)	0.3	2	0.1	2	0.12	0.12	3	1
Plant age (years)	2	> 20	3	> 20	5	5	20	20
Tillage practice	No-till (direct planting)	No-till (direct planting)	Harrowing	No-till (direct planting)	Plowing, harrowing	Plowing, harrowing	Plowing, harrowing	No-till (direct planting)
Crop rotation	Yes (peanut, corn)	Yes (oat, corn, bean, barley, soybean)	Yes (oats, radish, corn)	Yes (oat, corn, bean, barley, soybean)	No	No	No	No
Cover crop	Spontaneous vegetation	Cover crop mix	Crop residues	Cover crop mix	Nov 2023 – soybean; Feb 2024 – buckwheat, fodder radish, vetch; Aug 2024 – oat; Nov 2024 – sunn hemp (few plants); Mar 2025 – buckwheat”	Spontaneous vegetation (clover, vetch, hairy beggarticks)	Spontaneous vegetation, vetch	None
Fertilization	Sheep and cattle manure (each, 600 kg/ha)	20 (N), 75 (P₂O₅), 50 (K₂O) kg/ha	Poultry manure (60 kg/ha)	20 (N), 75 (P₂O₅), 50 (K₂O) kg/ha	Turkey and poultry manure (6 t/ha), phosphate and potassic rock powder (200 kg/ha), hydrolyzed fish (marine fish), sugarcane molasses, citric acid, and saccharides (80 mL/ha)	12 (N), 30 (P₂O₅), 30 (K₂O) kg/ha + 18 (N), 45 (P₂O₅), 45 (K₂O) kg/ha.	Sheep and turkey manure (each, 600 kg/ha)	None
Frequency of fertilization	Once per season	Twice per season	Once per season	Twice per season	Once per year	Twice per year	Every 2 years	-
Liming	No	Yes (lime according to soil analysis)	No	Yes (lime according to soil analysis)	No	Yes	Yes (lime 5 t/ha)	Yes (lime 2.5 t/ha)
Disease, pest & weed control	Lemon, vinegar, detergent	Chlorothalonil (720 g/ha), bixafen (63 g/ha), prothioconazole (88 g/ha), trifloxystrobin (365 g/ha). Spinetoram (12 g/ha). Clethodim (240 g/ha)	Neem oil (400 L/ha), cow urine	Chlorothalonil (720 g/ha), bixafen (63 g/ha), prothioconazole (88 g/ha), trifloxystrobin (365 g/ha). Spinetoram (12 g/ha). Clethodim (240 g/ha)	Bordeaux mixture (200 mL/ha), calcium polysulfide 200 mL/ha). Azadirachtin (400 mL/ha). Mowing	Mancozeb (3 kg/ha), oxadixyl + mancozeb (2.5 kg/ha), folpet (1.5 kg/ha), thiophanate-methyl (0.7 kg/ha), potassium phosphite (1.5 kg/ha), difenoconazole (0.12 L/ha), sulfur (3 kg/ha), pyraclostrobin + metiram (2 kg/ha), iprodione (1.5 L/ha). Surfactant (50 mL/ha). Mowing, herbicide	Bordeaux mixture (20 L/ha)	Glyphosate (5 L/ha)
Disease/disease frequency	Monthly	According to crop monitoring	Weekly	According to crop monitoring	According to crop monitoring	Weekly	At flowering	Every 3 months
Irrigation method	Manual irrigation (daily)	No	No	No	No	Yes (drip irrigation), according to the weather	No	No
Erosion control	Cover crops	Yes (no till, contour sowing, water retention basins)	No	Yes (no till, contour sowing, water retention basins)	Cover crops	Cover crops	Cover crops	No
Inoculant	No	*Bradyrhizobium* sp.	No	*Bradyrhizobium* sp.	*Bacillus subtilis*	No	No	No

Following collection, the samples were immediately transferred into sterile plastic bags, stored in coolers with ice, and subsequently refrigerated at 4 °C. Within 24 to 48 h, the samples were transported to specialized laboratories for molecular and chemical analyses.

### Survey among the farmers

Concurrently with soil collection, structured interviews were provided to the farmers. The interviews included pre-formulated questions designed to obtain contextual information on management history, input usage, and farmer practices. The questionnaires were left with the farmers for completion and later retrieval (Table 1).

### Ethics statement

This study involving human participants (survey) was approved by the Research Ethics Committee of the Midwestern State University of Parana (Comitê de Ética em Pesquisa da Universidade Estadual do Centro-Oeste – UNICENTRO, Brazil), in accordance with Brazilian National Health Council Resolution No. 466/2012. All participants were informed about the objectives of the study and provided informed consent prior to participation. Participant anonymity and confidentiality were ensured.

### Soil chemical properties analysis

Chemical analyses of the soil samples were performed at the Soil and Plant Nutrition Laboratory of the Midwestern State University of Paraná, Brazil. The analytical procedures followed the protocol described by Pavan et al.^36^. The following parameters were quantified: pH in CaCl₂ (0.01 mol L⁻¹) and in water, exchangeable aluminum (Al³⁺), potential acidity (H⁺ + Al³⁺), and concentrations of calcium, magnesium, potassium, and phosphorus, the last of which extracted using the Mehlich-1 method. Microelements (Fe, Cu, Mn and Zn) were also quantified.

For sulfate-sulfur, extraction was carried out using 0.01 mol L⁻¹ calcium phosphate solution as described by Cantarella and Prochnow^37^. The concentration of sulfate-sulfur was determined by the turbidimetric method according to Vitti and Suzuki^38^.

### Amplicon sequencing analysis

Soil DNA was extracted using the Qiagen DNeasy PowerSoil™ (QIAGEN^®^ - Catalog no. 47014) according to the manufacturer’s protocol. The DNA concentration was further verified with the NanoDrop spectrophotometer.

Amplicon PCR for bacteria was performed using the primers 341 F/785R (V3–V4 within the hypervariable regions of the 16 S rRNA gene)^39^, and for the fungi the ITS1-ITS2 primers were used. The forward primer sequence was V3 (CCTACGGGNGGCWGCAG) and the reverse was V4 (GACTACHVGGGTATCTAATCC). For fungi, our target was the ITS1 and ITS2 region^40^. The forward primer sequence was (TCCGTAGGTGAACCTGCGG) and the reverse was (GCTGCGTTCTTCATCGATGC), added with the overhang adapter (Illumina Co., USA). Index PCR and library construction were performed using S502-S522 as the forward primer and N712-N716 (Illumina, Inc.). After all PCR the target bands were cut and cleaned before move to the subsequent step, and the library was normalized at 4 µM. Then, the amplicon sequencing was performed using Illumina PE 300 bp at Cancer Research Institute (IPEC), Guarapuava, PR, Brazil. The sequences are deposited in the DNA Database of Japan (https://www.ddbj.nig.ac.jp/index-e.html*)*, BioProject accession number PRJDB35416, and BioSample metadata accession numbers SAMD00899141–SAMD00899204.

Bioinformatic analysis of the sequence data was performed using QIIME2-2023^41,42^, which was used to calculate relative abundance (RA) and microbial richness (Number of Operational Taxonomic Units – OTU, and Shannon-Wiener index). The OTUs clusters were defined by a 97% identity threshold, and the datasets were submitted to rarefaction analysis before estimate the diversity. The quality filtering was applied at a Phred score ≥ Q20. The taxonomy base analysis for the 16 S genes (bacteria) was accessed by the Silva database (https://www.arb-silva.de*)*, and the Unite database (https://unite.ut.ee/) was used for ITS (fungi, 42). Relative abundance and diversity index were used to evaluate how the microbial communities in bean and grape soils are influenced by the location and management system. Differences in the relative abundance and diversity indices were assessed using analysis of variance (ANOVA) after checking data normality with the Shapiro-Wilk test. Using the Microbiome Analyst platform (https://www.microbiomeanalyst.ca), genetic features at the genus level were first selected through Hierarchical Feature Engineering (HFE) prior to machine-learning analysis. Random Forest classification was then applied using management system as the experimental factor, with 5,000 trees grown and 14 predictors randomly tested at each split, with randomness enabled. Model performance was internally evaluated using the out-of-bag (OOB) error estimation implemented in the platform. This approach was used to evaluate whether group of treatments, such as location and management system, exhibited distinguishable microbial features.

The RA of bacteria was added at phylum level and that of fungi at class level, using the average values. Tukey test was used to calculate the significance among the RA. The predicted metabolic functions were analyzed using bacterial data at the species level via FAPROTAX^43^ and PICRUSt^44^. The Venn diagram was generated based on the counts for each group using a plotting library (matplotlib-venn). The final diagram visually represents the overlap (shared OTUs) and exclusivity (unique OTUs) of bacterial and fungal taxa between bean and grape microbiomes as determined by ITS amplicon sequencing.

### Relationship between soil chemical analysis and soil microbial communities

The relationship between soil chemical properties related to fertility and the microbial communities was analyzed using Pearson’s correlations for individual variables and the Non-Metric Multidimensional Scaling (NMDS). This last one was conducted using the values of soil chemical data and the number of OTUs within the soil microbial communities as variables, employing the ggplot2, ggrepel, and vegan packages in the statistical software RStudio 1.4. The fungal community data from similar plots were used to calculate NMDS, with the predicted metabolic functions performed with PICRUSt.

## Results

### Farmer-reported management

Key management differences with potential impacts on soil microbiota were observed between systems and crops. Organic systems relied on organic amendments such as manure, rock powders, hydrolyzed fish, and molasses, applied at lower frequencies, while conventional systems used synthetic fertilizers at higher rates, often combined with liming (Table 1). Disease and pest control in organic fields relied on permitted inputs (Bordeaux mixture, calcium polysulfide, azadirachtin, neem oil, and traditional homemade preparations) and mechanical methods (mowing), whereas conventional systems applied multiple synthetic fungicides, herbicides, and insecticides with higher frequency.

Tillage practices diverged notably: organic grapes were established with plowing and harrowing, whereas one conventional grape site adopted no-till; in both cases, soil management was restricted to vineyard establishment. Common bean systems were predominantly no-till, with harrowing in one organic field; in all cases, soil management was performed annually at crop establishment. Cover crop diversity was higher in organic grapes and absent in one conventional grape site; in beans, organic systems used spontaneous vegetation or crop residues, while conventional systems employed planned cover crop mixtures and, in some cases, structural erosion control measures. Microbial inoculants were restricted to organic grapes (*Bacillus subtilis*) and conventional beans (*Bradyrhizobium* spp.).

### Soil chemical properties

Comprehensive chemical analyses revealed marked differences among soil management systems (Table 2). Organic soils displayed elevated phosphorus (P) and aluminum (Al) contents, whereas conventional soils were characterized by higher pH, organic matter, and Ca levels. Key macronutrient and micronutrient concentrations, as well as cation exchange capacities (CEC), further distinguished the four systems (Organic, Conventional, Bean, Grape).

**Table 2:** Chemical properties of the soil (0–10 cm depth) under two cultivation systems and two types of plants.

Treatment	pH	Organic matter	*P*	K^+^	Ca^2+^	Mg^2+^	H + Al	Al^3+^	CEC
	CaCl_2_	g dm^− 3^	mg dm^− 3^	cmol_c_ dm^− 3^					
Organic	5.20	73.7	25.7	0.9	10.8	3.5	6.45	0.2	15.5
Conventional	5.99	83.2	16.1	1.2	15.7	2.3	3.8	0.06	19.2
Bean	5.33	89.9	19.9	0.6	11.8	3.6	6.22	0.19	16.2
Grape	5.86	67.0	21.9	1.4	14.8	2.2	4.02	0.06	18.4
Treatment	Fe	Cu	Mn	Zn	BS	m	K^+^	Ca^2+^	Mg^2+^
			mg dm^− 3^		%				
Organic	34.4	2.36	17.1	10.9	69.9	1.43	4.1	50.0	15.8
Conventional	35.6	2.92	14.2	12.0	83.1	0.31	4.97	68.5	9.6
Bean	28.8	1.07	6.2	11.5	71.8	1.35	2.93	53.0	15.9
Grape	41.2	4.21	25.1	11.4	81.2	0.4	6.14	65.5	9.6

### Soil microbial community

#### Microbial diversity

**Fig. 1:**
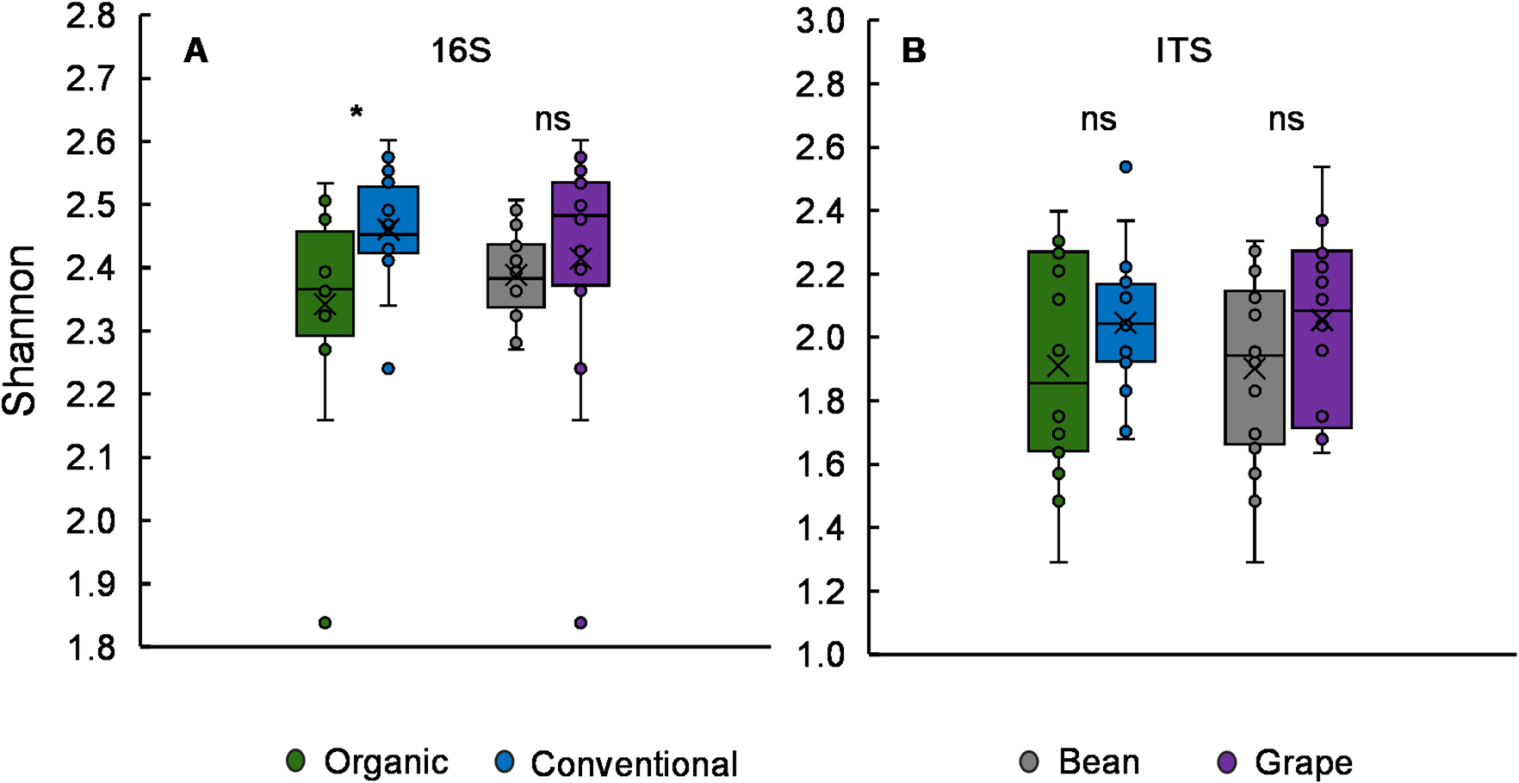
Bacterial (**A**) and fungal (**B**) richness represented by the number of Shannon-Wiener indexes in two cultivation systems and two types of plants. Error bars represent standard errors (SE), * indicates significant differences between the systems within the same plant type (*p* < 0.05); ns indicates non-significant difference.

Shannon index-based alpha diversity analysis revealed that the management system significantly impacted bacterial diversity (16 S, *p* = 0.0211), with conventional plots harboring higher diversity (Fig. 1A). No significant differences were observed by crop type (*p* = 0.6183 for 16 S; *p* = 0.1208 for ITS [fungi]). Fungal diversity responded less to management or crop, suggesting a greater resilience of fungal communities to these external factors (Fig. 1B).

#### Microbial community composition

**Fig. 2:**
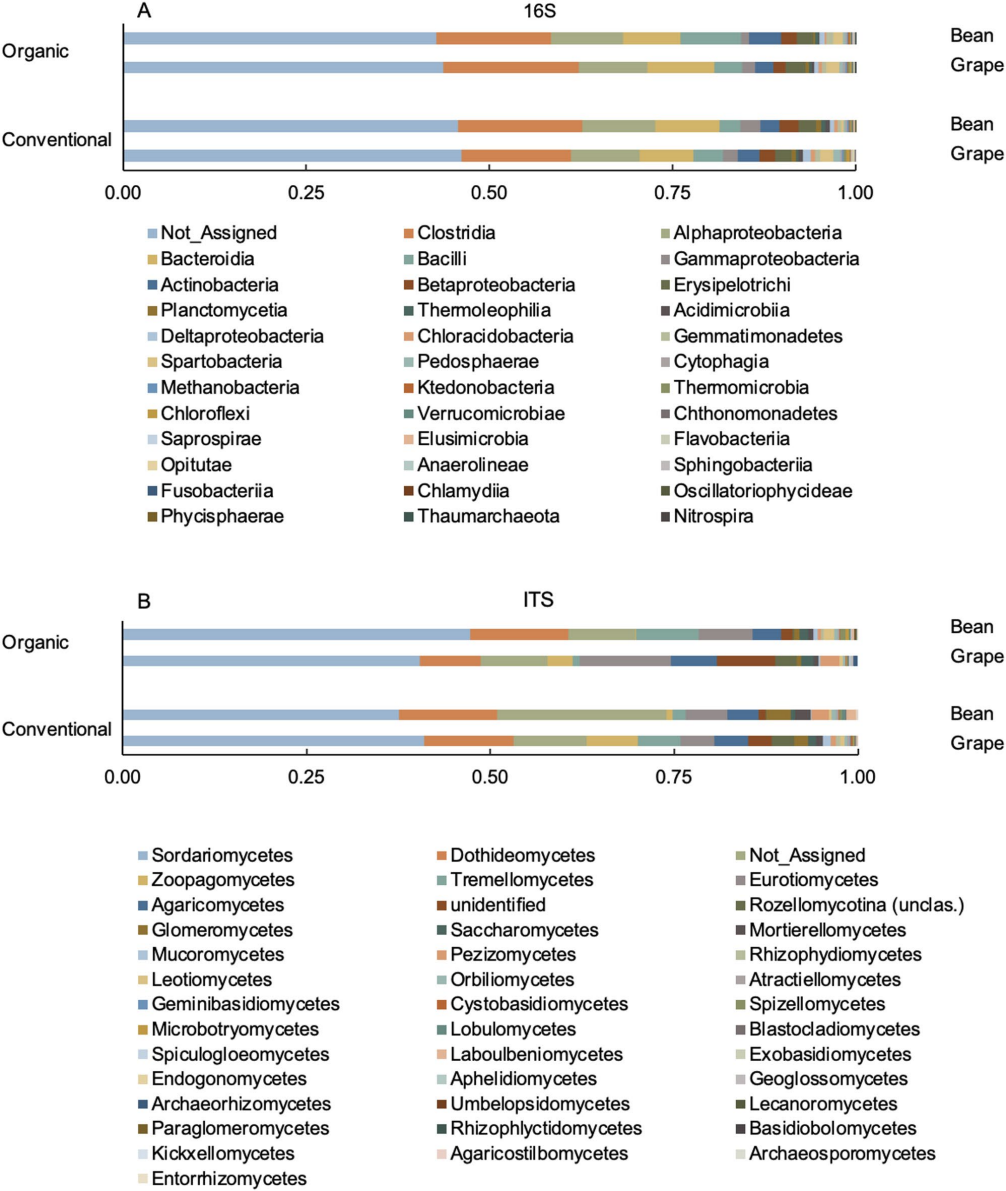
Distribution of relative abundance of bacterial classes (16 S-A) and fungal orders (ITS-B) under two cultivation systems and two types of plants.

Bacterial taxonomic profiling based on relative abundance (RA) revealed that *Clostridia*, *Alphaproteobacteria*, *Bacteroidia*, and unassigned taxa together accounted for more than 75% of the total bacterial community across all treatments (Fig. 2A). *Gammaproteobacteria* and *Acidimicrobiia* were comparatively more abundant in conventional soils. Fungal communities were dominated by *Sordariomycetes*, *Dothideomycetes*, *Zoopagomycetes*, *Tremellomycetes*, *Eurotiomycetes*, and unassigned taxa, with smaller but consistent contributions from *Agaricomycetes*, *Glomeromycetes*, and *Saccharomycetes* (Fig. 2B). Relative abundance among fungal taxa showed clearer contrasts between management systems: *Zoopagomycetes* and *Glomeromycetes* were more abundant under conventional management, whereas *Eurotiomycetes* and *Saccharomycetes* predominated in organic soils.

#### Discriminatory taxa and microbial bioindicators

**Fig. 3:**
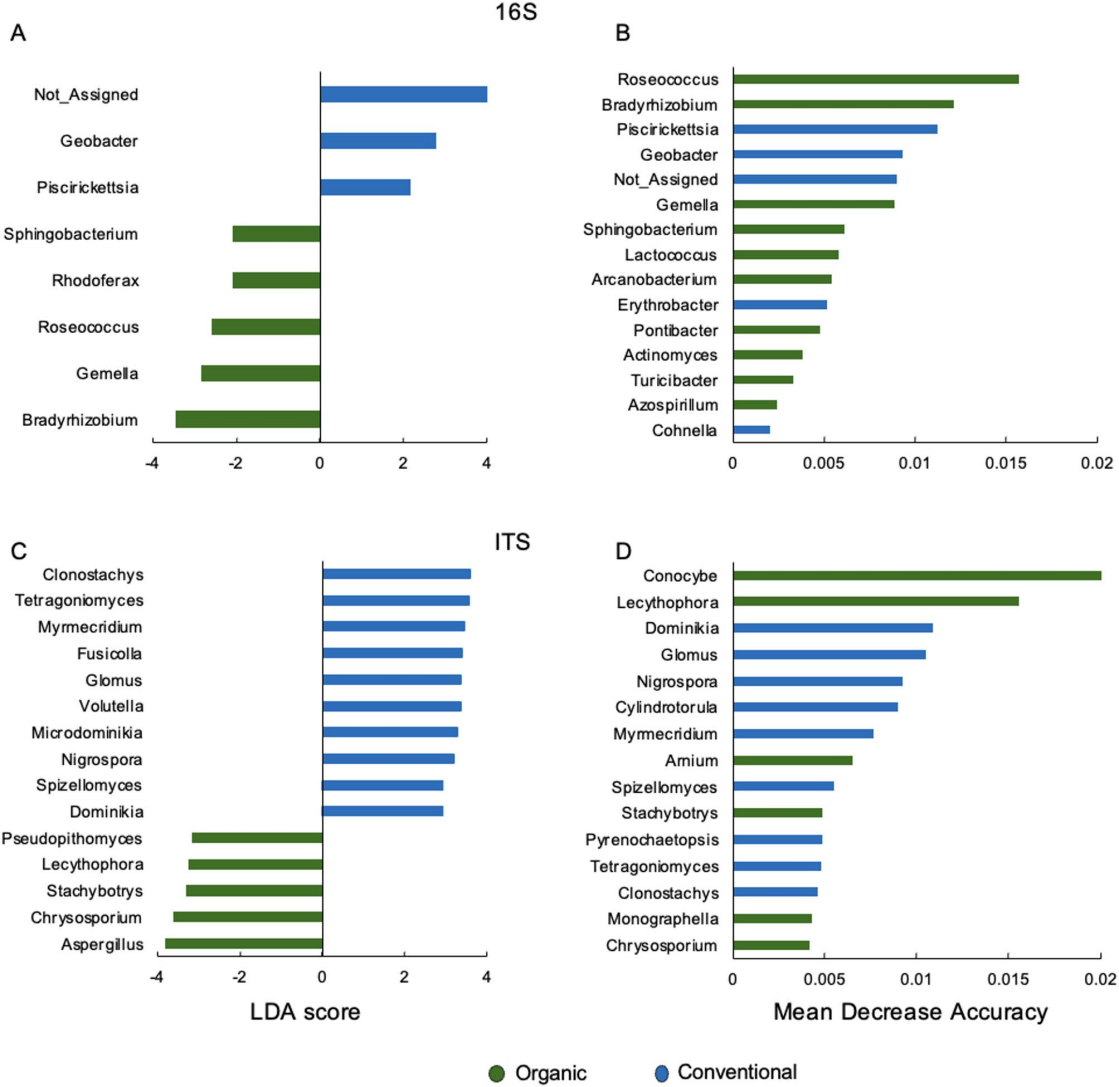
Classification level discriminant analysis (LDA) – A and B, and Random Forest Assessment – C and D. Part (**A**) shows the bacterial and (**B**) the fungal biomarkers with statistical difference for the genera. The colors of the legends represent the strength of the impact, and the length of the dots the scores size, which corresponds to the impact size of the taxa with significant differences.

Machine-learning analyses identified distinct bacterial and fungal taxa that discriminated between soil management systems (Fig. 3). For bacteria (16 S), LEfSe analysis revealed *Bradyrhizobium* and *Roseococcus* as biomarkers of organic soils, whereas *Geobacter* was associated with conventional soils (Fig. 3A). For fungi (ITS), LEfSe showed *Aspergillus* and *Chrysosporium* enriched under organic management, while *Trichoderma*, and *Peziza* were associated with conventional soils (Fig. 3B). Random Forest classification corroborated these patterns: *Roseococcus*, and *Bradyrhizobium* were ranked as the most important bacterial predictors of organic soils (Fig. 3C). For fungi, Random Forest identified *Conocybe* and *Lecythophora* as predictors of organic soils, whereas *Dominikia* and *Glomus* were stronger predictors of conventional soils (Fig. 3D). Overall, both methods consistently highlighted *Bradyrhizobium* and *Roseococcus* as bacterial and *Lecythophora* as fungal hallmarks of organic management.

**Fig. 4:**
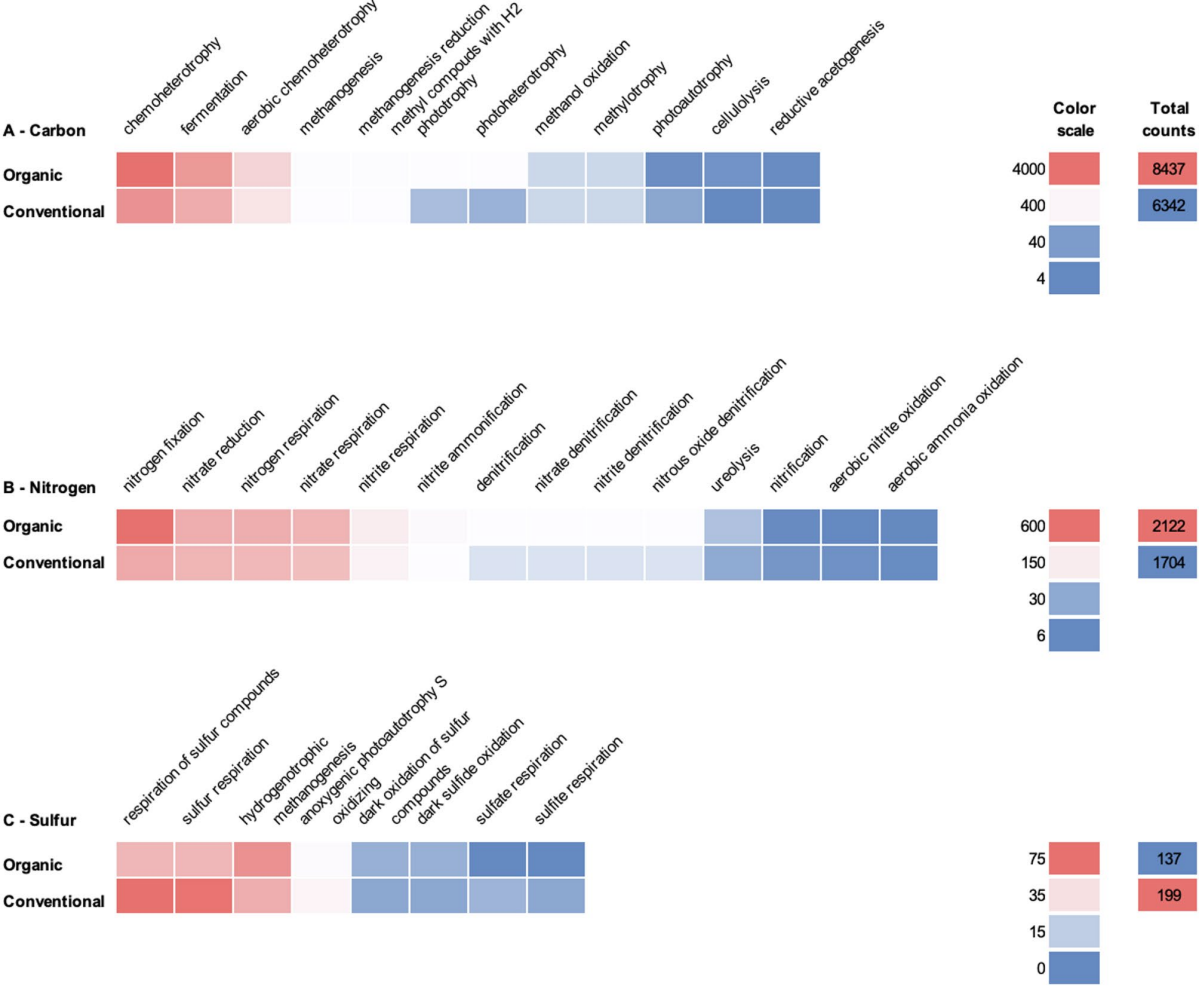
Predicted metabolic functions from the bacterial community among the cultivation systems, representing the Nitrogen (**A**), Carbon (**B**) and Sulfur (**C**) cyclings. The colors represent the number of OTUs linked to these functions.

Predicted functional profiling revealed a higher representation of bacterial operational taxonomic units (OTUs) associated with nitrogen and carbon metabolism in organic compared with conventional soils, whereas sulfur-related functions were more represented in conventional soils. For carbon metabolism (Fig. 4A), organic soils showed the largest difference, with 8440 OTUs versus 6342 in conventional soils, mainly associated with chemoheterotrophy, fermentation, and aerobic chemoheterotrophy. For nitrogen cycling (Fig. 4B), organic soils harbored 2122 OTUs versus 1704 in conventional soils, with marked enrichment in nitrogen fixation, nitrate reduction, and nitrate respiration. For sulfur metabolism (Fig. 4C), conventional soils exhibited higher overall representation (199 OTUs) than organic soils (137 OTUs); within this pathway, conventional soils were linked to respiration of sulfur compounds, and sulfur respiration. These results highlight contrasting bacterial functional potentials between management systems.

#### Core microbiome characteristics

Analysis identified distinct core microbiomes for each management system (Supplementary Fig. 1). Organic soils were characterized by a greater number of, but less consistently distributed, bacterial and fungal families; certain bacterial families (Bacillaceae, Gemellaceae, Sphingomonadaceae) were unique to organic management, while Rhizobiaceae was specific to conventional management. Bean cultivation, in particular, was associated with a broader range of core bacterial families. Conventional soils were dominated by Nectriaceae, Didymellaceae, and Cladosporiaceae, whereas organic soils showed higher prevalence of Chaetomiaceae, Mortierellaceae, and Helotiaceae. Notably, organic management supported a greater number of exclusive fungal families compared with conventional farming. In the crop comparison, bean samples were mostly associated with Nectriaceae and Didymellaceae, while grape samples showed a greater prevalence of Mycosphaerellaceae and Pleosporaceae.

### Relationship between microbial community and soil chemical properties

**Fig. 5:**
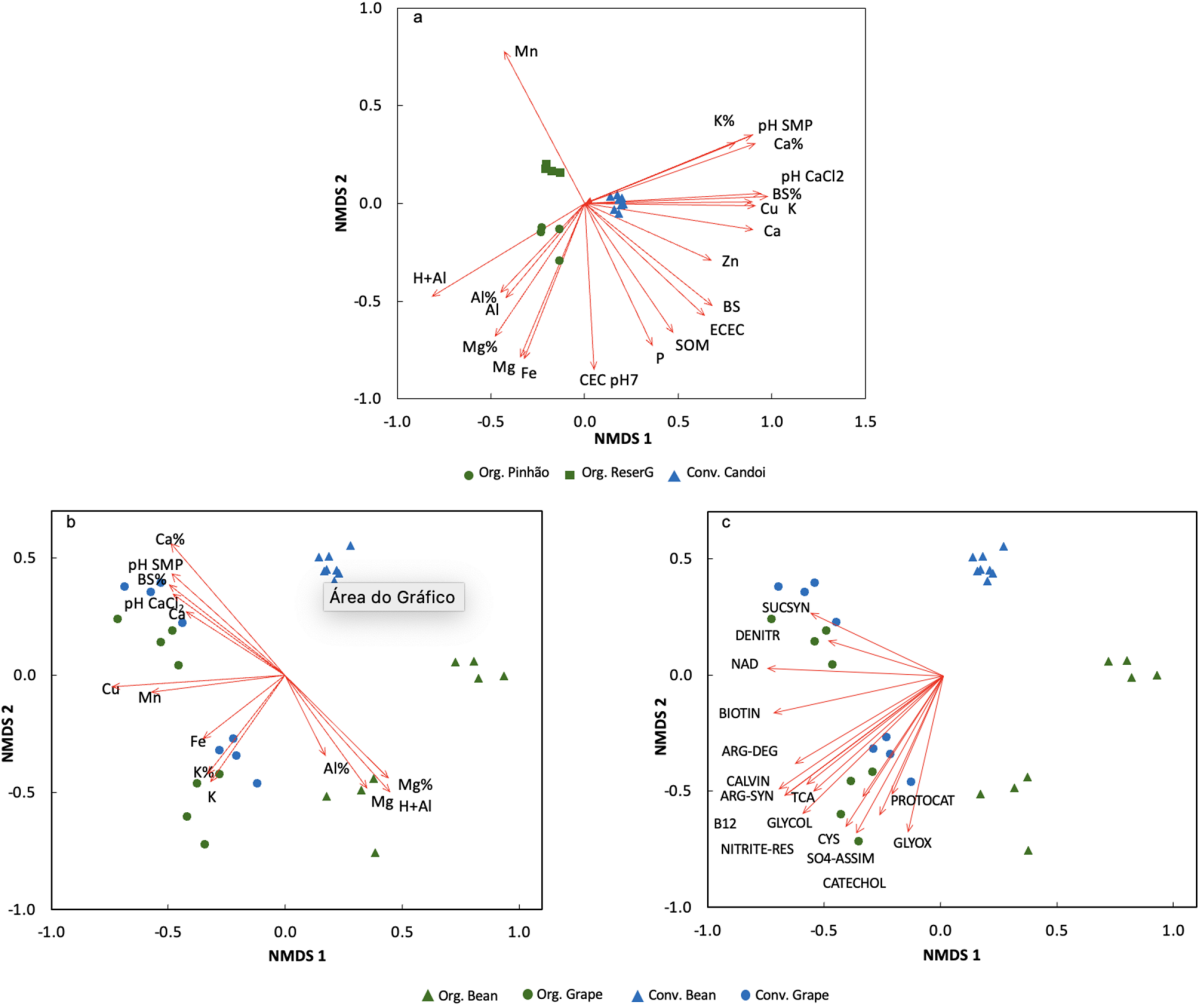
Non-metric multidimensional scaling (NMDS) plots of bacteria in Bean soils (**A**), fungi in both plants (**B**) and the relationship between fungal community and the predicted metabolic functions (C) in both plants.

The NMDS ordination revealed that bacterial community structure was strongly shaped by both soil management and associated soil chemical property (Fig. 5). Several chemical variables—specifically pH (SMP and CaCl₂), Ca²⁺, K⁺, BS%, Ca, Zn, and Cu—were positively correlated with the NMDS axes and clustered with samples from conventional systems (Fig. 5A). In contrast, Al³⁺, H + Al, Mg, Mn, and Fe were more closely associated with organic soil samples. This pattern indicates that variations in soil fertility and acidity are key drivers of bacterial community structure in bean-growing systems, underlying the differences observed between organic and conventional management.

NMDS ordination of fungal (ITS) communities showed a moderate but discernible separation by management system, most notably within grape soils, where samples (circles) clustered tightly according to management type (Fig. 5B). Bean samples (triangles) were more dispersed, reflecting greater variation in fungal communities across replicates. Chemical parameters such as Ca²⁺, pH (SMP and CaCl₂), and BS% were positively correlated with conventional samples, whereas Al% (Al³⁺), Mg²⁺, and H + Al aligned with organic treatments. These associations underscore the selective influence of soil chemical profiles, modulated by both management and crop, in shaping fungal community composition.

Moreover, the NMDS analysis of predicted metabolic pathways and fungal community further differentiated organic and conventional management (Fig. 5C). Most functional vectors—including those for nitrification, gallate degradation I pathway, and succinyl-CoA biosynthesis—aligned with conventional communities. Conversely, pathways such as enterobactin biosynthesis and creatinine degradation were more prevalent within organic treatments.

Pearson’s correlation analysis revealed significant associations between soil chemical variables, microbial diversity indices, and specific bacterial taxa (Supplementary Fig. 2 A). Shannon diversity was negatively correlated with Ca²⁺ saturation (%). At the taxon level, *Clostridium butyricum*, *Plesiomonas shigelloides*, and *Stenotrophomonas* showed similar responses, being positively correlated with Mg²⁺ and Ca²⁺ saturation (%) and Al³⁺, but negatively correlated with K⁺ saturation (%).

For fungi (Supplementary Fig. 2B), richness (observed features) and Shannon diversity were positively correlated with Al³⁺. Several taxa displayed consistent associations with soil variables. For instance, *Chloridium aseptatum*, *Penicillium senticosum*, and *Preussia persica* were positively correlated with multiple elements, including Mg²⁺, Al³⁺, potential acidity (H + Al), and Ca²⁺ saturation (%), while showing negative correlations with soil pH (SMP), effective cation exchange capacity (ECEC), and K⁺ saturation (%). In contrast, taxa such as *Edenia gomezpompae* and *Keithomyces acicularis* were negatively correlated with H + Al but positively correlated with pH (SMP), Ca²⁺, ECEC, and K⁺ saturation (%).

### Distribution of fungal and bacterial OTUs

Venn diagrams revealed management- and crop-dependent differences in microbial communities (Supplementary Fig. 3). For bacteria (16 S), 265 OTUs were shared, with 55 unique to organic and 44 unique to conventional soils. For fungi (ITS), 530 OTUs were shared, while 244 and 152 were exclusive to organic and conventional soils, respectively. When comparing crops, bacterial communities shared 263 OTUs, with 59 and 42 uniquely associated with grape and bean, respectively. Fungal communities showed 476 OTUs in common, while 277 and 173 were exclusive to grape and bean. Overall, organic management harbored a greater number of unique bacterial and fungal OTUs than conventional management.

## Discussion

### Management practices and soil chemical properties

The divergent soil chemical properties observed here (Table 2) can be traced directly to the contrasting nutrient-management philosophies adopted by farmers (Table 1). These practices created distinct soil chemical environments that directly influenced microbial community assembly. Organic fields received regular poultry, turkey, sheep, and cattle manures—inputs that supply not only readily mineralizable phosphorus but also large quantities of organic carbon that fuel microbial respiration, thereby favoring copiotrophic and functionally specialized microbial groups. During decomposition, microbially produced organic acids donate protons to the soil solution, driving pH downward and releasing Al³⁺ from clays and oxides through ligand-promoted dissolution, a chemical shift known to constrain microbial assembly by excluding pH- and metal-sensitive taxa. Long-term studies indicate that poultry-litter or organic manure applications can increase soil pH or ameliorate acidity, often decreasing exchangeable Al concentrations rather than increasing them; typical applications have been shown to raise surface-soil pH by 0.4–1.3 units and reduce exchangeable Al^17–20^.

The farmer-reported management confirms that just one organic farmer applied lime during the study period. As a result, acidity generated by manure mineralization was unbuffered. This explains the elevated Al levels and lower base saturation observed in organic soils relative to conventional soils. Liming is well known to precipitate Al as Al-hydroxides and to replenish Ca²⁺ and Mg²⁺ on exchange sites. These changes expand the range of microbial taxa able to persist under less acidic conditions. A meta-analysis analyzed 30 experiments across 48 locations and found that acid soil management practices can increase yield by 67% relative to control, with lime + phosphorus achieving the highest yield increase^21^, matching the higher pH and Ca stocks maintained by the conventional farmers who incorporated CaCO₃ according to soil-test recommendations. Trace-metal distributions closely reflected management practices and likely contributed to microbial selection. Conventional vineyards that applied Bordeaux mixture or Cu-oxychloride fungicides showed two- to three-fold higher extractable Cu and Zn than their organic counterparts, consistent with patterns observed in European vineyards with long-term copper use^22^. Copper is known to accumulate in the topsoil due to its tendency to form stable complexes in near-neutral pH soils, leading to persistence where repeated applications occur^22^. In contrast, acidic organic soils often contained more bioavailable Mn, a micronutrient abundant in animal manures and more soluble at low pH. This metal availability further contributes to shifts in microbial community composition.

Studies show that decreasing soil pH enhances DTPA-extractable Mn and alters bacterial communities, a process likely at play in manure-amended bean plots^18^. These observations collectively illustrate how amendment choices, liming frequency, and pesticide type influence soil chemistry, ultimately shaping the plant nutrient environment and microbial ecology. A study by Bakari et al.^23^ also reported the effect of liming on the soil microbiome and N cycle. They concluded that co-application of lime and inoculation has potential of increasing nodulation and N fixation in strongly acidic soils, which further corroborates the importance of the input and its influence on the soil microbial community.

Field tillage history and management intensity may have also contributed to these patterns in soil properties and microbiota. No-till systems, commonly adopted in conventional bean fields and at certain vineyard sites, are documented to enhance soil organic matter, aggregate stability, and base saturation, consistent with the higher organic matter and nutrient retention observed in these systems despite synthetic input reliance^24^, conditions that promote greater microbial habitat heterogeneity and bacterial richness. Reduced soil disturbance favors bacterial richness due to the maintenance of microhabitat variability. In contrast, organic fields displayed pronounced variability in management age, some converted only recently, and others managed organically for many years, introducing heterogeneity in chemical and microbial properties. This variation is echoed in β-diversity patterns, as newly organic fields may retain microbial legacies from past conventional inputs, while older fields display transitions toward manure-driven enrichment and more acid-tolerant, copiotrophic assemblages^25,26^. Thus, inputs, tillage regime, and management duration interact to produce the distinct soil chemical and microbial signatures observed across systems.

### Effects of agricultural management on soil microbial communities

#### Microbial diversity and community composition

Higher bacterial α-diversity observed in conventional plots (Fig. 1A) likely stems from elevation of soil pH through systematic liming. From an ecological perspective, soil pH functions as a primary environmental filter that constrains microbial niche space and community assembly. Hartmann et al.^27^ found contrasting results in their study, where organic farming increased richness, decreased evenness, reduced dispersion and shifted the structure of the soil microbiota when compared with conventionally managed soils under exclusively mineral fertilization. This was largely attributed to the quality of the organic inputs. However, large-scale studies consistently report pH as the strongest determinant of bacterial richness, with diversity increasing almost monotonically from strongly acidic to neutral soils^28^. Mechanistically, pH neutralisation broadens the range of bacterial guilds able to thrive by reducing proton stress and aluminum toxicity, while increasing calcium availability and aggregate stability enhances microhabitat diversity^24^. These conditions expand ecological opportunities for diverse bacterial lineages such as Actinobacteria. In contrast, fungal diversity showed little variation across the pH gradient (Fig. 1B), consistent with global findings that fungi are adapted to a wider pH and nutrient range due to features like chitinous cell walls and extensive hyphal networks^29^. These adaptations allow fungi to maintain richness under both acidified and neutral conditions^30^.

Venn analysis revealed that organic management and crop regime produced a greater pool of unique bacterial and fungal OTUs, consistent with evidence that diverse organic amendments and reduced chemical disturbance foster ecological niche differentiation. Crop identity also strongly structured microbial communities, reflecting root-mediated recruitment and litter quality effects^31^. These findings align with niche-based theory, in which plant–soil feedbacks and resource heterogeneity promote compositional turnover without necessarily increasing total richness^27,31^.

#### Discriminatory taxa and microbial bioindicators

Machine-learning analysis pinpointed *Bradyrhizobium* and *Roseococcus* as markers of organic management systems (Fig. 3). *Bradyrhizobium*, a symbiotic nitrogen fixer, is commonly enriched in legume-dominated organic systems, while *Roseococcus* is a less-studied but notable inhabitant of organic-rich, acidic soils. Their presence suggests organic management selects for functionally specialized groups. Organic soils’ combination of lower pH, elevated P, and higher exchangeable Al acts as a selective filter, supporting stress-tolerant species like *Bradyrhizobium*—consistent with recognized adaptation strategies for acidic, chemically stressed soils^18^. *Roseococcus* may also be resilient under these conditions, a hypothesis warranting additional research. Copiotrophic fungi such as *Aspergillus* and *Conocybe* dominated organic systems, thriving on readily decomposable manure-derived carbon. Functionally, organic soils were enriched in nitrogen fixation and respiration genes, consistent with manure-driven stimulation of the nitrogen cycle^24^. Conversely, conventional soils were enriched in sulfur metabolism pathways, paralleling sulfur application histories.

Beyond taxonomic differentiation, predicted functional profiles derived from FAPROTAX and PICRUSt analyses provided additional insight into the ecological implications of management-driven microbial selection. Organic soils showed enrichment of pathways related to nitrogen fixation and organic matter decomposition, consistent with previous reports of enhanced microbial activity and nutrient cycling under organic management^8,9,24,25^. In contrast, conventional systems were associated with sulfur metabolism pathways, reflecting fertilization practices and fungicide histories commonly reported in intensively managed agroecosystems^11,33^. These functional patterns broadly aligned with the taxonomic shifts observed; however, predicted functions should be interpreted cautiously given the inherent limitations of inference-based approaches^43,44^.

### Linking soil chemistry, microbial structure, and management systems

Soil pH emerged as a master driver of microbiome assembly, consistent with previous research by Lauber et al.^32^; community composition shifts tracked pH gradients and exchangeable Al, with Acidobacteria and metal-tolerant fungi predominant in low-pH, high-Al organic soils, and Al-sensitive taxa flourishing where pH was higher^28^. Increased P availability in organic soils selectively favored phosphate-solubilizing taxa such as Bacillaceae and Sphingomonadaceae, matching findings from global fertilizer trials^33^. In conventional fields, sulfur-fertilized soils are enriched for sulfur-oxidizing microbes, a trend consistent with microbiome responses to fungicide histories. Distinct core microbial assemblages for each management system suggest limited functional redundancy and vulnerability to rapid management changes, highlighting the importance of gradual amendment strategies for system stability^34^.

### Implications for sustainable soil management and agroecosystem resilience

Targeted liming in organic fields may alleviate Al toxicity and increase microbial diversity without negating compost benefits, as evidenced in acid Ultisol remediation^35^. Matching manure P inputs with crop needs could curb eutrophication risks. Meanwhile, lowering sulfur fungicide use in conventional vineyards could promote a more functionally robust soil microbiome. These results reinforce the central role of management—through soil testing, tailored amendments, and microbiome monitoring—in optimizing both fertility and belowground biodiversity.

This study is limited by the number of sampled sites and by its regional scope. Thus, extrapolation to other agroecosystems should be made with caution.

In summary, our findings demonstrate that organic and conventional management systems shape soil microbial communities in contrasting ways. Organic soils were characterized by bacterial biomarkers such as *Bradyrhizobium* and *Roseococcus*, despite exhibiting lower overall bacterial diversity compared with conventional soils. Fungal communities, in turn, were more strongly associated with conventional management, with only a few taxa, such as *Lecythophora*, linked to organic soils. These results highlight that management practices act as selective filters, favoring specific microbial taxa rather than uniformly enhancing diversity, and underscore the importance of integrating microbial indicators with soil chemistry and farmer-reported management practices to better understand the ecological outcomes of different agricultural systems.

## Supplementary Information

Below is the link to the electronic supplementary material.

Supplementary Material 1

## Data Availability

The metagenomic sequence data generated during this study are publicly available in the DNA Data Bank of Japan (DDBJ) under the accession number PRJDB35416. The data can be accessed through the DDBJ Search portal at https://ddbj.nig.ac.jp/search. The same project is also be available at the NCBI BioProject under the same accession number.
