# Supplementary material for: Exploration of soil microbial community: a comparative study between organic and conventional systems in perennial and annual crops
Source: Sci Rep. 2026 May 14;16:22024. doi: 10.1038/s41598-026-49298-1 (PMC13365416; doi:10.1038/s41598-026-49298-1)
Supplement: Supplementary file 1 — Supplementary Material 1 [file 41598_2026_49298_MOESM1_ESM.docx]

**Exploration of Soil Microbial Community: A Comparative Study Between Organic and Conventional Systems in Perennial and Annual Crops**

Jackson Kawakami^1*^; Mateus Oliveira Gomes^2^; Paulo Roberto Da-Silva^1^; André Freire Cruz^2^

^1^Midwestern State University of Paraná, UNICENTRO, Paraná, Brazil; ^2^Kyoto Prefectural University, Kyoto, Japan.

^*^ Corresponding author

E-mail: jkawakami@unicentro.br


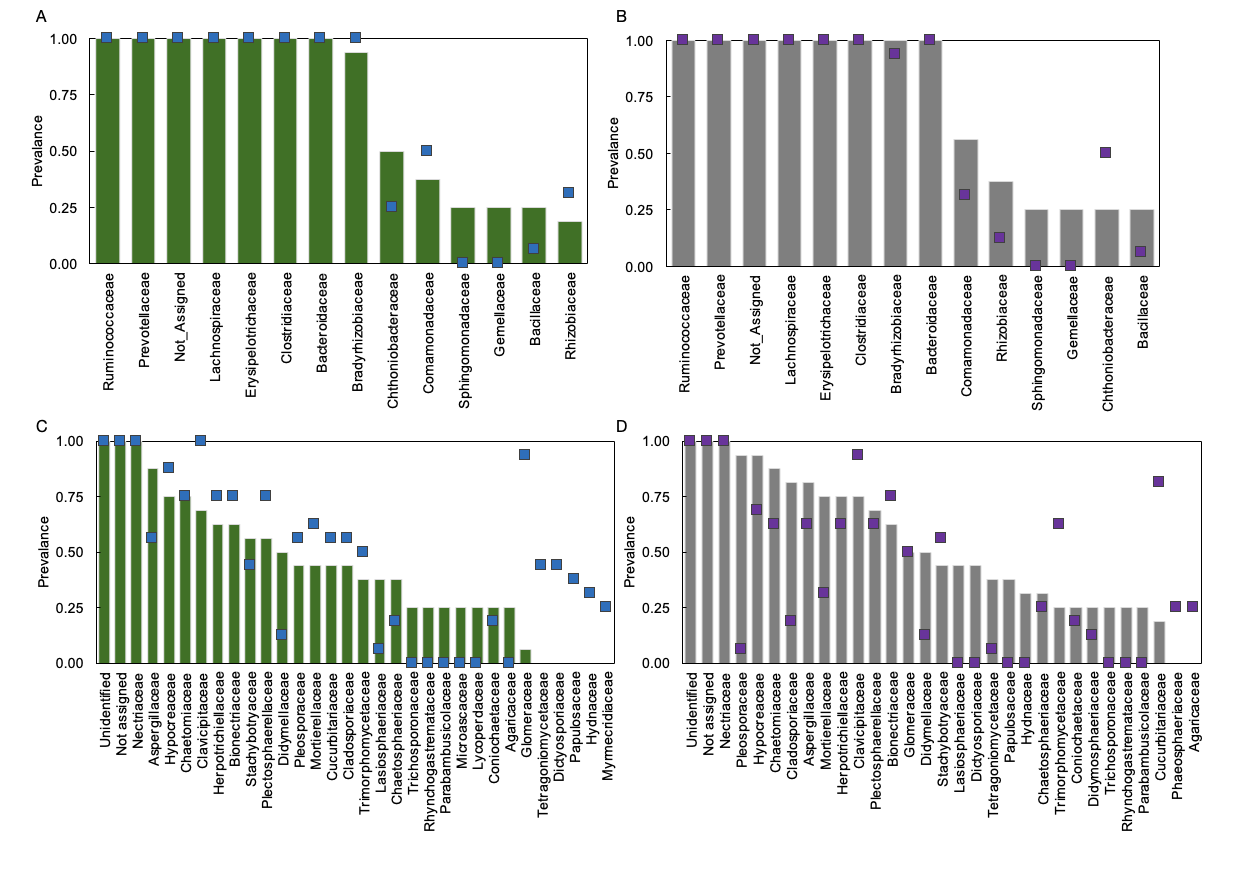


Supplementary Figure 1. Bacterial (A-B) and fungal (C-D) biomarkers within two management systems (organic: bars, conventional: squares, A, C) and crops (bean: bars, grape: squares, B, D).

A


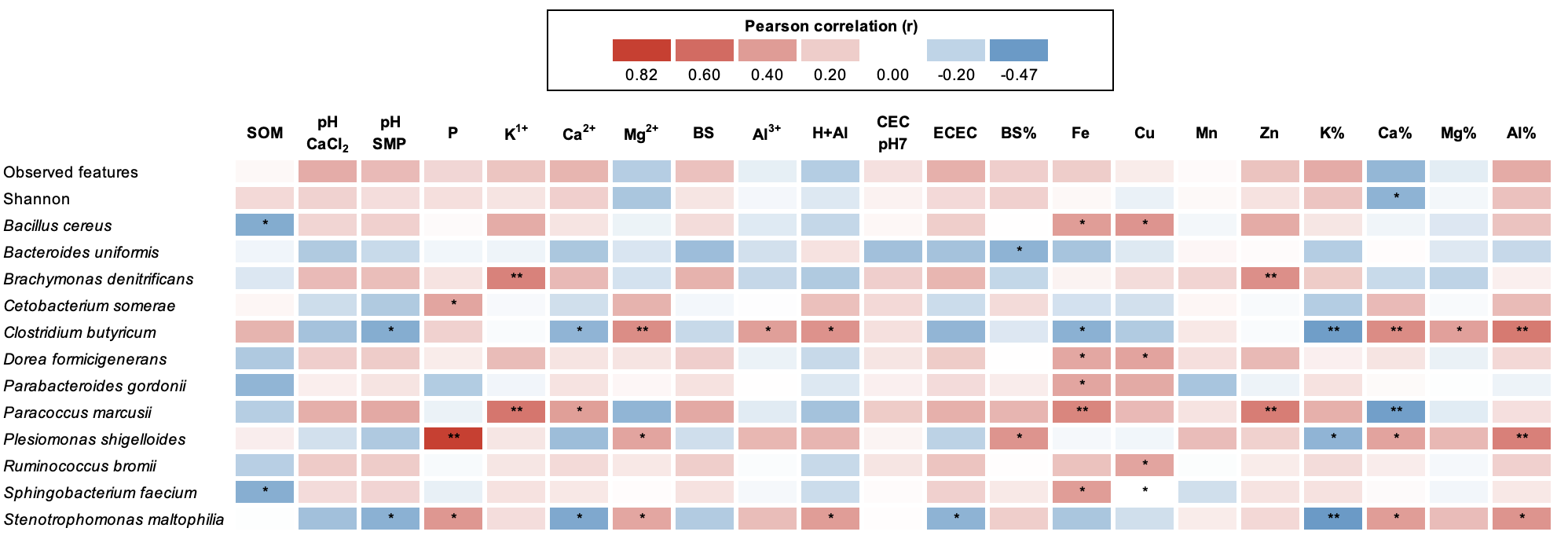


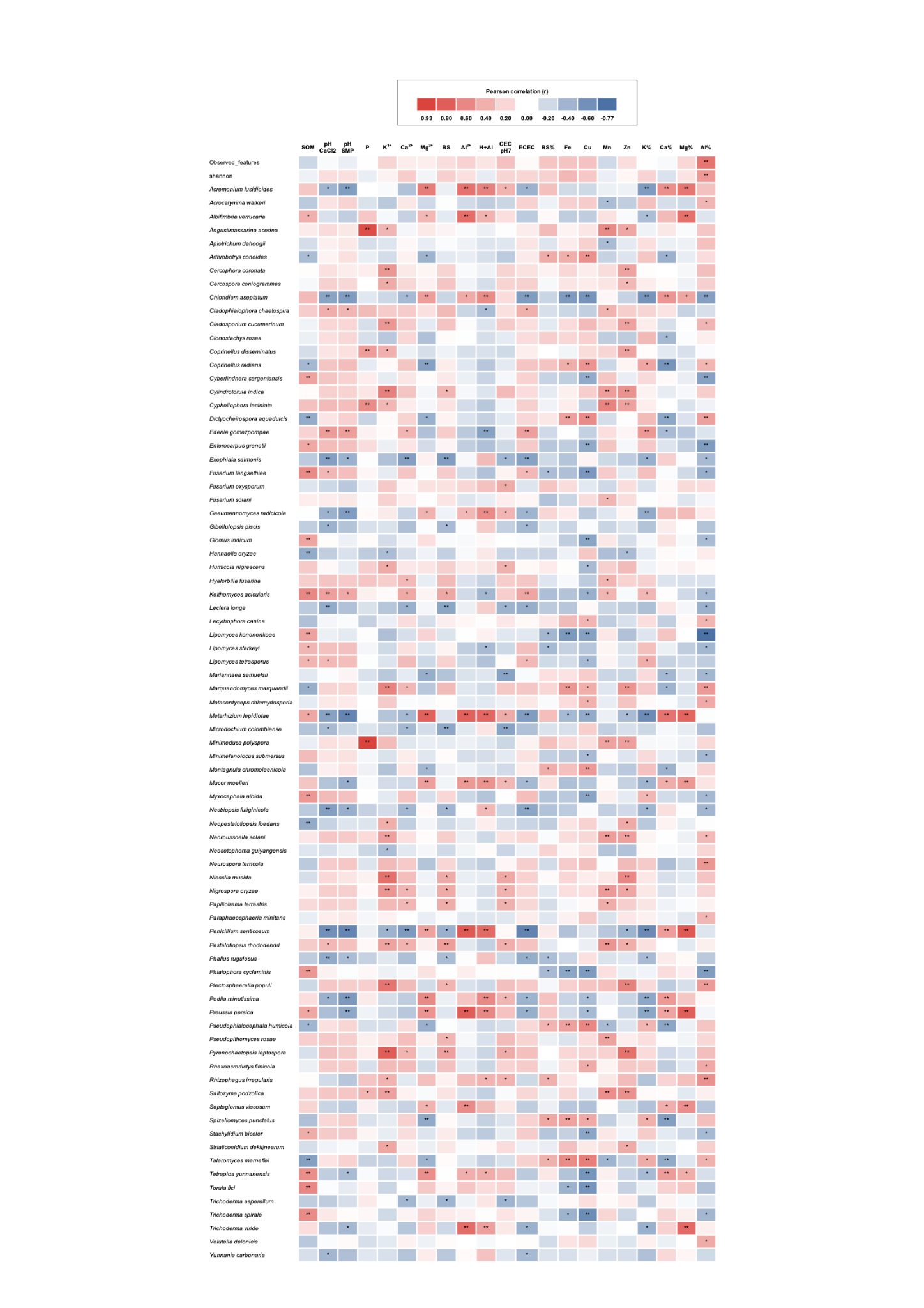
B

Supplementary Figure 2. Pearson correlation between the bacteria (16S-A) and fungal (ITS-B) features and soil chemical properties.

**
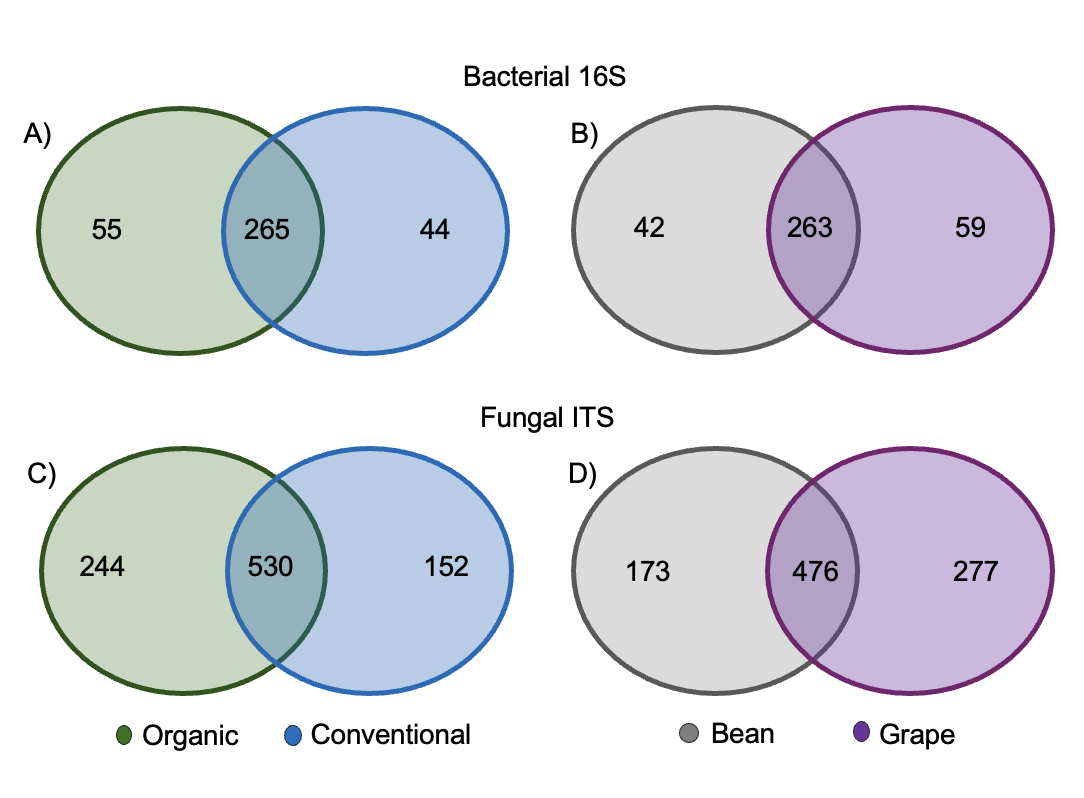
**

Supplementary Figure 3. Venn diagrams showing shared and unique bacterial (16S, A-B) and fungal (ITS, C-D) OTUs between management systems (organic, conventional, A-C) and crops (bean, grape, B-D).
